# Extracellular Vesicles from *Escherichia coli* Strains of the Gut Microbiota Trigger Hepatic Antioxidant and Anti-Lipogenic Effects via the Gut-Liver Axis in Healthy Neonatal Rats

**DOI:** 10.3390/nu17193066

**Published:** 2025-09-25

**Authors:** Sergio Martínez-Ruiz, Josefa Badia, Laura Baldoma

**Affiliations:** 1 Departament de Bioquímica i Fisiologia, Facultat de Farmàcia i Ciències de l’Alimentació, Universitat de Barcelona, 08028 Barcelona, Spain; sergio_martinez_ruiz@ub.edu (S.M.-R.); josefabadia@ub.edu (J.B.); 2 Institut de Biomedicina de la Universitat de Barcelona (IBUB), 08028 Barcelona, Spain; 3 Microbiota Intestinal, Institut de Recerca Sant Joan de Déu (IRSJD), Santa Rosa 39-57, 08950 Esplugues de Llobregat, Spain

**Keywords:** intestinal microbiota, bacterial extracellular vesicles, postbiotics, gut-liver axis, liver metabolism, inflammation, oxidative stress, early-life

## Abstract

**Background**: The gut-liver axis is essential for maintaining liver physiology, with the gut microbiota playing a central role in this bidirectional communication. Recent studies have identified microbiota-derived extracellular vesicles (EVs) as key mediators of inter-organ signaling. This study explored the impact of EVs from two beneficial *Escherichia coli* strains, the probiotic EcN and the commensal EcoR12, on hepatic metabolism and oxidative stress in healthy neonatal rats. **Methods**: EVs were administered orally during the first 16 days of life, and blood and liver samples were collected on days 8 and 16. **Results**: The results demonstrated that EVs significantly reduced intestinal permeability, as evidenced by decreased plasma zonulin levels. In the liver, EVs enhanced redox homeostasis by downregulating *CYP2E1* and upregulating key antioxidant genes (*SOD1*, *CAT*, *GPX*). Furthermore, the treatment shifted liver metabolism toward an anti-lipogenic profile by inducing fatty acid oxidation genes (*PPARA*, *CPT1A*) and suppressing genes involved in de novo lipogenesis (*SREBP1C*, *ACC1*, *FASN*, *CNR1*). Importantly, markers of hepatic inflammation remained unchanged, indicating the safety of the intervention. In vitro experiments using human HepG2 cells supported these findings, further validating the antioxidant and metabolic effects of the EVs. **Conclusions**: Our results underscore the role of microbiota-derived EVs as important mediators of hepatic metabolic programming in healthy individuals via the gut-liver axis and highlight their potential as therapeutic postbiotic agents for management of fatty liver diseases.

## 1. Introduction

The intestine, particularly its resident microbial community, plays a crucial role in regulating human health. The gut microbiota supports a wide range of essential physiological functions, including nutrient absorption and metabolism, immune system development, and the maintenance of intestinal barrier integrity [1,2]. Moreover, it actively participates in complex communication networks that link the gut to distant organs throughout the body [3,4].

The composition of the gut microbiome is shaped by a multitude of factors, such as genetics, diet, and lifestyle-related environmental exposures. Any disturbance of the intestinal environment can lead to an imbalance in the microbial community, a condition known as dysbiosis. Increasing evidence suggests that dysbiosis not only contributes to gastrointestinal disorders but also exerts pathophysiological effects on extraintestinal tissues [5,6].

The intestinal mucosa forms a critical barrier to face the huge number of microbes and luminal antigens in close proximity. The intestinal barrier comprises three structurally and functionally distinct layers that work coordinately to preserve gut integrity. The outermost layer is the mucus layer, which is rich in mucins secreted by goblet cells. This layer not only provides a habitat for commensal bacteria but also acts as a physical coating that prevents direct interaction between microbes and the underlying epithelial monolayer [7]. Underneath the mucus layer lies the intestinal epithelium, formed by specialized cells connected by tight intercellular junctions, which seal the paracellular space to impede passage of luminal bacteria to the underlying tissue. The deepest layer is the immune system located in the lamina propria, which shapes and mounts defensive responses against pathogens that succeed in crossing the epithelial boundary. Together, these three layers work synergistically to regulate the transport of molecules and compounds from the gut to the liver and subsequently into the systemic circulation. Under healthy conditions, the epithelial tight junctions remain intact, permitting the absorption of water and nutrients while effectively preventing harmful intestinal contents from entering the bloodstream and internal organs [8,9]. However, dysregulation of the gut microbiota can compromise the gut barrier, leading to a leaky gut characterized by increased intestinal permeability.

Since the gut microbiota’s access to the host is physically and chemically restricted by the intestinal barrier, microbiota–host communication primarily occurs through microbiota-secreted factors such as metabolites, bioactive molecules, and extracellular vesicles (EVs) [10,11]. EVs are released by all bacterial species and serve as a crucial tool for intercellular communication. These vesicles are nanosized, double-layered structures that originate from bacterial membranes and encapsulate various biomolecules found in the parent cells, including proteins, lipids, metabolites, and genetic material like DNA and RNA [12]. Numerous studies on probiotic and gut microbial strains have shown that EVs mediate microbiota functions by delivering functional molecules into host cells, thus influencing signaling mechanisms and cellular processes [10].

A substantial body of evidence now indicates that microbiota-derived EVs can cross cellular barriers and disseminate throughout the body [13]. Experimental support for the systemic distribution of microbiota EVs in healthy individuals has been obtained from studies using mouse models [14,15] as well as from blood samples of healthy human donors [16]. In this context, EVs released by the gut microbiota are emerging as key components of the communication networks linking the gut to distant organs [17,18].

The gut-liver axis refers to the bidirectional relationship between the gut, its microbiota, and the liver. The interaction is established primarily through the portal vein, which carries gut-derived products to the liver. By this route, metabolites such as short-chain fatty acids and EVs released by a balanced and healthy microbiota community can reach the liver and modulate hepatic immune and metabolic processes [19]. However, risk factors such as unbalanced diet, alcohol abuse, virus infection, or immune dysfunction disrupt the intestinal epithelial barrier facilitating the translocation of inflammatory microbial products that activate the immune system and cause liver dysfunction and injury [6,20]. Alterations in lipid and glucose metabolism, steatosis, oxidative stress, and inflammation are common traits in chronic alcoholic- and non-alcoholic fatty liver diseases [21,22]. In fact, increased intestinal permeability is a common hallmark of a wide range of metabolic and neurological disorders [6].

Given the critical involvement of intestinal dysbiosis in the development of chronic liver diseases, emerging therapeutic strategies are increasingly focusing on the gut, and in particular on the gut microbiome [22,23]. This includes innovative approaches utilizing next-generation probiotics and postbiotics [24,25]. Among postbiotics, EVs produced by beneficial gut bacteria are emerging as promising tools for intervention [26,27].

Previous studies from our group have provided evidence that EVs derived from probiotic and gut beneficial E. coli strains exhibit immunomodulatory and barrier strengthening effects in various models of epithelial and immune cells [10,28]. Furthermore, preclinical studies in rodent models of inflammatory bowel disease and rotavirus infection have shown that oral administration of EVs derived from these strains alleviates diarrhea, clinical symptoms, and inflammation [29,30]. Overall, our research has broadened the understanding of how probiotic and beneficial gut microbes modulate immune responses and safeguard intestinal homeostasis.

Here, we investigated whether EVs from microbiota-derived E. coli strains could modulate hepatic metabolism and inflammation through the gut-liver axis in healthy neonatal rats. EVs from the probiotic *Escherichia coli* Nissle 1917 (EcN) and the commensal EcoR12 were administered daily to healthy suckling rats during the first 16 days of life. This experimental approach has previously been shown to be a safe intervention for stimulating immunity and intestinal maturation during the neonatal period [31]. In the present study, we assessed the presence of EVs in liver samples and analyzed the impact of the intervention on intestinal permeability and hepatic gene expression related to inflammation, oxidative stress response and lipid metabolism. Additionally, we used the HepG2 hepatic cell line to confirm that direct stimulation of liver cells with EVs produced effects consistent with those observed in vivo.

## 2. Materials and Methods

### 2.1. Bacterial Strains and Isolation of Extracellular Vesicles (EVs)

The probiotic *E. coli* strain EcN was from Ardeypharm (GmbH, Herdecke, Germany) and the commensal *E. coli* strain EcoR12 was isolated from a fecal sample of a healthy human adult [32].

Bacterial EVs were isolated from culture supernatants following established protocols [33]. In summary, bacterial cultures were centrifuged to obtain cell-free supernatants, which were then passed through 0.22 µm filters (Merck Millipore, Burlington, MA, USA) to remove any remaining bacterial cells. The filtrates were subsequently concentrated using Centricon Plus-70 filters with a 100 kDa cutoff (Merck Millipore). EVs were pelleted by ultracentrifugation (150,000× *g* for 1 h at 4 °C), followed by two washes with phosphate-buffered saline (PBS), and finally resuspended in PBS. The EV samples were quantified by protein concentration using the Pierce BCA protein assay (Thermo Fisher Scientific, Barcelona, Spain). Additionally, vesicle quantification was determined by membrane labelling using the fluorescent dye FM4-64 (Thermo Fisher Scientific, Barcelona, Spain). This method ensured consistent vesicle amounts between EcN and EcoR12 preparations [31]. EV samples were tested for sterility and stored at −20 °C for future use.

### 2.2. Animals and Experimental Design

This study extends previous research that employed neonatal rats to assess the impact of interventions based on EVs derived from the probiotic EcN and the commensal EcoR12 on intestinal maturation and immunity. The methodology for animal housing, experimental design, and EV administration has been previously reported [31].

In brief, nine pregnant Lewis rats at gestational day 14 (G14) were obtained from Janvier Labs (Le Genest-St-Isle, France) and housed individually under controlled environmental conditions, including a 12-h light/dark cycle, regulated temperature and humidity, in a biosafety level 2 facility at the Animal Experimentation Unit (UEA), Diagonal Campus, University of Barcelona. All animals were provided with unrestricted access to water and fed the commercial AIN-93G diet (Teklad Global Diet 2014, Inotiv, Inc., West Lafayette, IN, USA) [34].

Rats were monitored daily until spontaneous delivery. After birth, pups were randomly allocated to three experimental groups, standardizing litter sizes to eight pups per dam. Sex distribution was maintained as balanced as possible, aiming for a 40–60% male-to-female ratio per litter. All pups had unrestricted access to maternal milk. EV administration was performed via oral gavage from postnatal day 2 through day 16. Dosages were adjusted by age: 2 µg of EVs per pup daily from day 2 to day 8, and 5 µg per pup from day 9 to day 16. Control pups received the same volume of PBS as vehicles.

Blood and tissue samples were collected on days 8 and 16. The methodology for animal euthanasia, biometric measurements, and organ collection were described in our previous study [31]. Blood was collected via cardiac puncture, processed to obtain plasma, and stored in aliquots at −20 °C. Liver tissues were snap-frozen in liquid nitrogen and stored at −80 °C for subsequent molecular and biochemical analyses.

We previously reported that treatment with EVs derived from either EcN or EcoR12 was not associated with adverse effects, as it did not alter body weight, growth-related parameters (Body Mass Index, Lee Index), organ weights, or stool consistency [31]. Values for body and liver weights across all control and experimental groups at days 8 and 16 are provided in Supplemental Figure S1.

All procedures involving animals were conducted following approval from the Ethics Committees for Animal Experimentation of both the University of Barcelona (CEEA-UB Ref. 169/20) and the Catalonian Government(DAAM, Ref. 11461), in accordance with the European Directive 2010/63/EU on the protection of animals used for scientific purposes.

### 2.3. Cell Culture and Stimulation Conditions

Human hepatocellular carcinoma cells (HepG2; 85011430, human liver, Epithelial, Merck, Madrid, Spain) were maintained in 75 cm^2^ flasks (ref. 3290; Corning Glass Works, Burlington, MA, USA ) at 37 °C in a 5% CO_2_ environment. The cells were cultured in DMEM/F12 medium (Merck, Madrid Spain ref. D8900–10 X 1 L), supplemented with 10% fetal bovine serum (FBS; Cultilab, Guadalajara, Mexico, ref. 0521–500) and 1% penicillin-streptomycin (Corning, Fisher Scientific, Inc. Barcelona, Spain, Cat. #30-002CI). Cell proliferation was monitored daily under an inverted optical microscope, and all procedures were conducted within a laminar flow hood. Once the cultures reached approximately 80% confluence, the cells were washed with PBS, detached from the flask using trypsin, and subsequently plated into appropriate plates, depending on the experiment. For cell viability assays, cells were transferred into 96-well plates and cultured for 24 h before the addition of EVs. For gene expression analysis, HepG2 cells were plated at a density of 2 × 10^5^ cells/mL in 12-well plates and maintained in culture until 85–90% of confluence. The cells were then exposed to EcN EVs or EcoR12 EVs at a concentration of 5 μg/mL for either 8 or 24 h. As a control, a parallel culture was maintained without bacterial EV treatment. Additionally, the impact of a higher concentration of EVs (50 μg/mL) on HepG2 monolayers was also evaluated. Following the incubation period, culture supernatants and cells were collected for ELISA and quantitative RT-PCR analyses, respectively.

### 2.4. Cell Viability Assays

Following treatment of HepG2 cells with EcN or EcoR12 EVs, cell viability was assessed using the MTT (3-(4,5-Dimethylthiazol-2-yl)-2,5-diphenyl tetrazolium bromide) assay as described previously [35]. The results were expressed as the percentage of cell survival relative to untreated control cells.

### 2.5. Gene Expression Analysis by Reverse Transcription—Quantitative PCR (RT-qPCR)

Liver tissue samples were processed using lysing matrix tubes (MP Biomedicals, Illkirch-Graffenstaden, France) and homogenized with the FastPrep-24 instrument (MP Biomedicals). Total RNA was extracted using the RNeasy^®^ Mini Kit (Qiagen, Madrid, Spain) according to the manufacturer’s instructions. RNA quantity and quality were evaluated with the Varioskan Lux multimode reader (Thermo Fisher Scientific, Barcelona, Spain). Quantitative PCR was performed using the ABI Prism 7900 HT system (Life Technologies, Madrid, Spain).

For hepatic gene expression analysis, cDNA was synthesized from isolated RNA using the TaqMan^®^ Reverse Transcription Reagents kit (Applied Biosystems, Darmstadt, Germany), following the supplier’s guidelines. Quantitative PCR was performed with TaqMan^®^ assays (Applied Biosystems, Foster City, CA, USA). The selected targets included genes associated with lipid metabolism (*SREBP1C*, *FASN*, *ACC1*, *PPARA*, *CNR1*), oxidative stress pathways (*CYP2E1*, *INOS*, *COX2*, *SOD1*, *CAT*, *GPX*), and inflammation (*TNFA*, *TGFB*, *IL12*). Gene expression levels were normalized against the housekeeping gene *GUSB*. The expression of genes related to glucose metabolic pathways (*G6PASE*, *PCK2*, *GCKL*) was assessed by the SYBR Green protocol using *GAPDH* as the reference gene. Information on primers and probes is provided in Supplemental Tables S1 and S2.

For gene expression analysis using the HepG2 human hepatoma cell line, reverse transcription was conducted using the High-Capacity cDNA Reverse Transcription Kit (Applied Biosystems, Foster City, CA, USA), and gene amplification was carried out using SYBR^®^ Green PCR Master Mix (Applied Biosystems) along with gene-specific primers targeting the same panel of genes as in the liver samples. The *TBP* gene served as the internal control. Primer sequences used in this assay are listed in Supplemental Table S2.

Relative gene expression was determined using the 2^−∆∆CT^ method [36], with results expressed as fold change relative to control conditions.

### 2.6. Quantification of Cytokines, LPS and Zonulin by Enzyme-Linked Immunosorbent Assays (ELISA)

For HepG2 cells, culture supernatants were collected after the indicated treatments, centrifuged at 10,000× *g* for 20 min at 4 °C, and stored at −80 °C until further analysis. The concentrations of secreted IL-8 and TNF-α were quantified using commercial ELISA kits (BD Biosciences, San Jose, CA, USA), following the manufacturer’s instructions.

Lipopolysaccharide (LPS) was quantified in liver extracts using the High-Sensitivity ELISA Kit for Lipopolysaccharide (Cloud-Clone Corp., Katy, TX, USA; Cat. No. HEB526Ge-96T), which displays a detection range spanning from 12.35 to 1000 ng/mL. Crude extracts were prepared from liver tissue using fresh lysis buffer provided by the same supplier (Cat. No. CC-IS007-1). Protein concentration was measured by the Pierce BCA protein assay.

Zonulin concentration was assessed in plasma samples using a commercial 96-well Rat Zonulin ELISA kit (MYBioSource, San Diego, CA, USA; Cat. No. MBS2606662), according to the manufacturer’s protocol. The range of detection spans from 1.56 ng/mL to 100 ng/mL.

### 2.7. Dot Blot Assay

The presence of EVs in liver samples was analyzed by dot blot on a nitrocellulose membrane. Liver extracts were prepared and quantified in terms of protein concentration as described for the ELISA-based measurement of LPS. A total protein amount of 20 µg was spotted on the membrane for each sample. The membrane was blocked for 30 min in EveryBlot blocking buffer ((Bio-Rad, Alcobendas, Madrid, Spain) Cat. 12010020) and then incubated overnight with rabbit polyclonal anti-*E. coli* OmpA antibody (Invitrogen, Waltham, MA USA, Ref. ZK4543559, 1/2000 dilution), mouse monoclonal anti-*E. coli* LPS antibody (Abcam, Cambridge, United Kingdom; Ref. AB55654, 1/2000 dilution) or mouse monoclonal anti-vinculin antibody (Santa Cruz Biotechnology, Dallas, Texas, USA, Ref. 73614, 1/10,000 dilution) in Tris-buffered saline (TBS)-Tween. After two washes in TBS-Tween, the membranes were incubated for 1 h with anti-rabbit or anti-mouse HPR-conjugated antibodies (dilution 1/10,000) in TBS-Tween. After two washes, the membranes were revealed by chemiluminescence using the Amersham ECL Prime Western detection reagent, Ref. RPN2236.

### 2.8. Lipid and Glucose Determination

Plasma glucose and triglyceride levels were determined using commercial colorimetric assay kits. Glucose concentration was measured with the Glucose Kit (Cat. No. 11229010, LINEAR Chemicals, S.L., Barcelona, Spain), while triglyceride levels were assessed using the Triglycerides Kit (Cat. No. 41032, Spinreact, Girona, Spain), following the manufacturers’ protocols. To measure TAG content in the liver, 50 mg of frozen tissue was homogenized in 500 μL of buffer. Then, methanol-chloroform-extracted lipids were dried under an N_2_ stream and dissolved in ethanol. TAG amount was quantified in the extracted lipids using the commercial assay kit.

### 2.9. Measurement of Enzyme Activities in Liver

Frozen liver samples were resuspended in buffer 50 mM Tris –HCl, pH 7·5, 4 mM EDTA, 50 mM NaF, 0·5 mM phenylmethylsulfonylfluoride (PMSF), 1 mM dithiothreitol and 250 mM sucrose (1:5, *w*/*v*) and homogenized in lysing matrix tubes (MP Biomedicals, Illkirch-Graffenstaden, France) using a Fast-Prep-24 equipment (MP Biomedicals). After centrifugation at 20,000× *g* for 30 min at 4 °C, the supernatant was used to determine alanine aminotransferase (ALT) and aspartate aminotransferase (AST) activities using colorimetric reaction-based kits (Cromatest, LINEAR Chemicals, S.L., Barcelona, Spain). Protein concentration was quantified by the Pierce BCA protein assay.

### 2.10. Statistical Analysis

All statistical analyses were conducted using SPSS software, version 22.0 (IBM Corp., Chicago, IL, USA). To assess data distribution and variance homogeneity, the Shapiro–Wilk and Levene’s tests were employed, respectively. When assumptions of normality and equal variance were met, group comparisons were performed using one-way analysis of variance (ANOVA), followed by Tukey’s post hoc test to identify specific group differences. In cases where data did not follow a normal distribution or showed unequal variances, the non-parametric Kruskal–Wallis test was applied, with Dunn’s post hoc test used for multiple comparisons. A *p*-value less than 0.05 was considered indicative of statistical significance. Data are presented as mean values ± standard error of the mean (SEM).

## 3. Results

### 3.1. Orally Administered EVs Reach the Liver While Enhancing Gut Barrier Integrity

**Figure 1:**
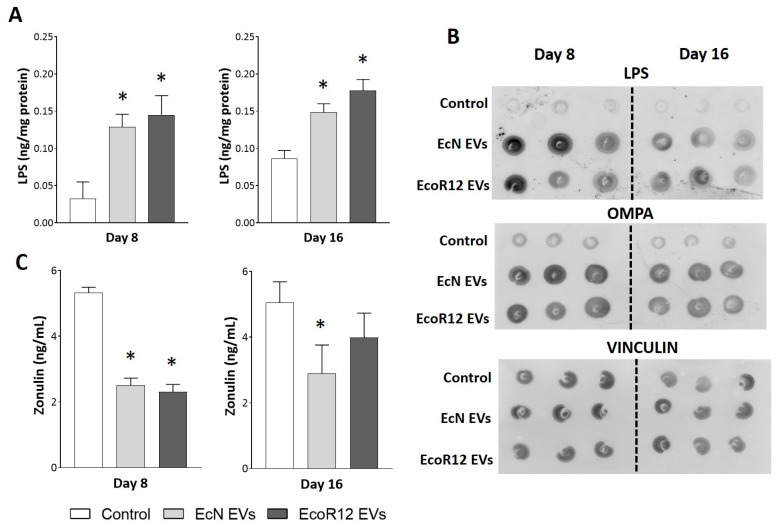
Detection of orally administered EVs in liver samples and their impact on plasma zonulin levels. Neonatal rats were administered PBS as the control or EVs of the indicated microbiota strains (from days 2 to 16 of life). By days 8 and 16, liver and plasma samples were collected. (**A**) The presence of bacterial EVs in the liver was assessed by means of LPS quantification by ELISA. (**B**) Representative dot blot assays of LPS, OMPA and vinculin as an endogenous control of loaded protein (20 µg protein). (**C**) Quantification of the intestinal permeability marker zonulin in plasma samples using ELISA. Statistical differences: * *p* < 0.05 compared to control (by post hoc Dunn’s multiple comparison test).

As evidence of the gut-liver axis function, we sought to prove that orally administered EVs reached the liver. First, the presence of EVs in the liver was indirectly assessed by quantifying LPS by ELISA in liver extracts obtained from tissue samples collected on days 8 and 16 (Figure 1A). Neonatal rats receiving daily EcN or EcoR12 EVs exhibited significantly higher LPS levels in the liver than control pups at both time points. In addition to its distribution via bacterial EVs, LPS can also be released as a soluble product during the turnover of intestinal Gram-negative bacteria. Therefore, quantification of LPS is not sufficient to prove the presence of bacterial EVs. To confirm that the elevated LPS levels detected in the liver of treated rats were associated with EVs, we performed dot blot assays in liver extracts (20 µg total protein) using specific antibodies against the *E. coli* outer membrane protein OmpA (Figure 1B). This protein is a typical transmembrane protein of the Enterobacteriaceae outer membrane that has been widely used as a marker of bacterial vesicles [16,37]. Dot blot immunodetection of LPS was performed in parallel as a control. The analysis revealed that livers from rats receiving treatment (EcN-EVs or EcoR12-EVs) exhibited a higher OmpA signal than that of control animals, while the intensity of the hepatic protein marker vinculin was similar in all groups (Figure 1B). A strong correlation was observed between hepatic LPS levels and OmpA signal across all experimental groups. Although detection of EVs was not based on specific-strain markers, the differences in hepatic LPS and OmpA content between control and treated animals may be attributed to the orally administered EVs.

The passage of gut microbiota EVs through the paracellular route and their subsequent entry into the bloodstream are facilitated by increased intestinal permeability. Previous studies from our group have shown that oral gavage of EcN or EcoR12 EVs in neonatal rats upregulates the expression of occludin in the small intestine by day 8 of life [31]. This finding suggested that EVs could contribute to barrier protection, particularly in the neonatal period, where the immaturity of the small intestine is linked to enhanced paracellular permeability [38]. However, the impact of EcN or EcoR12 EVs on intestinal permeability had not been assessed until now. Here, we quantified plasma zonulin levels as a marker of barrier dysfunction [39,40]. Neonatal rats administered with EVs from either the probiotic EcN or the commensal EcoR12 exhibited significantly lower circulating zonulin levels than control animals on day 8, indicating that EV treatment enhanced intestinal barrier integrity (Figure 1C). By day 16, statistical significance between experimental groups was only observed for the EcN-EV treated group.

Overall, the results confirmed that orally administered EVs reach the liver through the portal vein as part of the normal gut-liver communication network, rather than as a consequence of increased paracellular passage induced by the treatment.

### 3.2. Interventions with EcN and EcoR12 EVs Reduce Liver Inflammation and Improve the Antioxidant Response in Neonatal Rats

**Figure 2:**
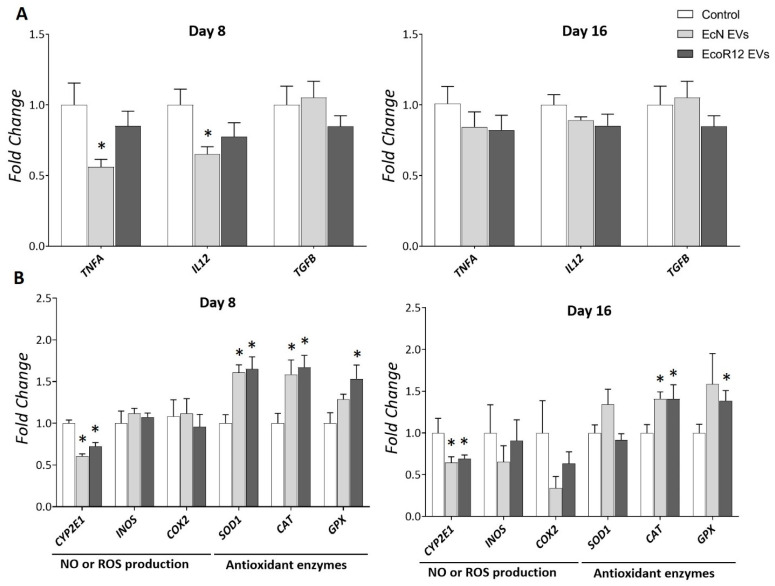
Regulation of genes involved in liver inflammation (**A**) and oxidative stress balance (**B**) by orally administered EcN and EcoR12 EVs. Neonatal rats were administered PBS as the control or EVs of the indicated gut microbiota strains (from days 2 to 16 of life). By days 8 and 16, liver samples were collected and processed for RNA isolation. The transcription levels of the indicated genes were quantified by RT-qPCR using *GUSB* as the reference gene. Relative mRNA levels in the interventional groups were calculated with respect to the CON group (expression value set to 1). Results were expressed as ±SEM (*n* = 8 animals/group and time point). Statistical differences: * *p* < 0.05 compared to CON group (by post hoc Dunn’s multiple comparison test). Abbreviations: *TNFA*, tumor necrosis factor-alfa; *IL12*, interleukin-12; TGFB, transforming growth factor-β; *CYP2E1*, cytochrome P-450 2E1; *COX2*, cyclooxygenase-2; *INOS*, inducible nitric oxide synthase; *SOD1*, superoxide dismutase; *CAT*, catalase; *GPX*, glutathione peroxidase.

LPS induces hepatic oxidative stress and inflammation, and elevated LPS levels have been associated with the onset and progression of chronic inflammation-related diseases [41,42]. In the liver, the inflammatory response induced by LPS predominantly involves resident macrophages, known as Kupffer cells, which release pro-inflammatory cytokines that exacerbate liver damage [42,43]. Since, in our model, the elevated LPS levels detected in the liver of treated rats were associated with the administered EVs, we assessed the effects of the interventions with EcN and EcoR12 EVs on liver inflammation by analyzing the expression of genes encoding TNF-α (*TNFA*) and IL-12 (*IL12*) (Figure 2A). By day 8, none of the treatments led to an increase in the mRNA levels of these pro-inflammatory cytokines compared to control. Moreover, administration of EVs from the probiotic EcN significantly downregulated the expression of *TNFA* and *IL12* at this time point. This finding aligns with previous reports on the anti-inflammatory effects of this probiotic and its EVs in various inflammation models [29,35,44,45]. After 16 days of treatment, *TNFA* and *IL12*mRNA levels in the EV-treated groups were comparable to those in the control group. Moreover, interventions based on EcN or EcoR12 EVs did not alter the expression of transforming growth factor β (TGF-β), even after 16 days of treatment. This pleiotropic cytokine plays a role in both suppressive and inflammatory immune responses, depending on the pathophysiological condition. In the liver, high levels of TGF-β contribute to liver injury, causing inflammation, fat accumulation, and fibrosis [46,47]. Overall, the results presented in Figure 2A indicate that EV administration did not induce adverse effects on liver inflammation.

Oxidative stress and inflammation are closely related pathophysiological processes that can be triggered by common mediators through TLR signaling pathways. Moreover, these processes mutually influence each other. Since microbiota EVs enclose various microbial-associated molecular patterns (MAMPs) that are recognized by host Toll-like receptors (TLRs), we analyzed the expression of genes encoding enzymes involved in the antioxidant response, namely superoxide dismutase (*SOD1*), catalase (*CAT*) and glutathione peroxidase (*GPX*), as well as enzymes that generate reactive oxygen or nitrogen species, such as cytochrome P-450 2E1 (*CYP2E1*) and inducible nitric oxide synthase (*INOS*), respectively. The study also included the gene encoding the inducible cyclooxygenase isoform (*COX2*). In addition to its role in synthesizing the inflammatory mediator prostaglandin E_2_ from arachidonic acid, COX2 enzyme is an important source of ROS generation. Gene expression was assessed by RT-qPCR in liver samples collected on days 8 and 16 (Figure 2B).

By day 8, interventions with EcN or EcoR12 EVs significantly upregulated mRNA levels of the antioxidant enzymes *SOD1*, *CAT*, and *GPX* while downregulating *CYP2E1* expression. Except for *SOD1*, this expression pattern was maintained after 16 days of treatment. At this time point, *SOD1* mRNA levels were higher in the group treated with EVs from the probiotic EcN compared to the control and EcoR12-EV-treated groups, although the differences did not reach statistical significance. Oral administration of EVs did not influence *INOS* or *COX2* expression. However, long-term treatment with EcN EVs tended to reduce *COX2* mRNA levels (Figure 2B), a finding consistent with their anti-inflammatory properties.

The results showed that in healthy animals, beneficial gut bacteria may positively modulate the hepatic antioxidant response through their released EVs.

### 3.3. EcN and EcoR12 EVs Regulate Hepatic Lipid Metabolism via the Gut-Liver Axis by Promoting Lipid Oxidation and Inhibiting Lipid Synthesis

**Figure 3:**
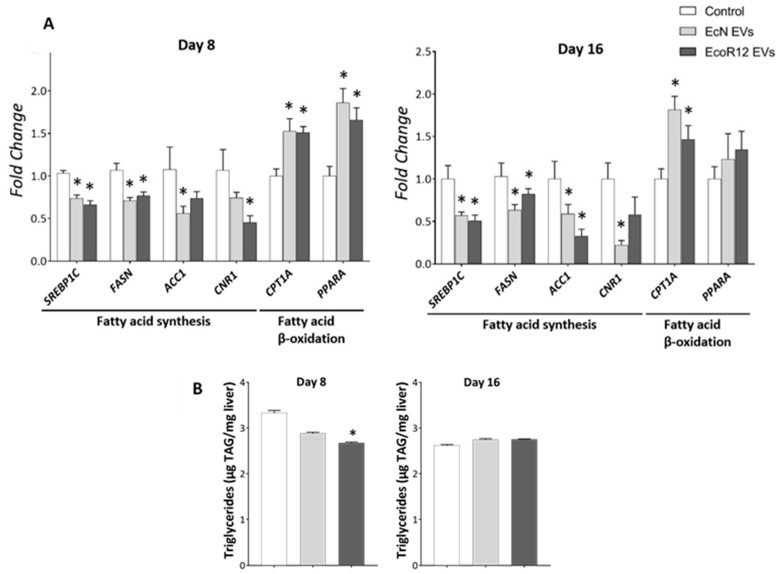
Regulation of hepatic genes involved in fatty acid synthesis and oxidation by orally administered EcN and EcoR12 EVs. Neonatal rats were administered with PBS as the control or EVs of the indicated gut microbiota strains (from days 2 to 16 of life). By days 8 and 16, liver samples were collected and processed for RNA isolation and lipid extraction. (**A**) The transcription levels of the indicated genes were quantified by RT-qPCR using *GUSB* as the reference gene. Relative mRNA levels in the interventional groups were calculated with respect to the CON group (expression value set to 1). (**B**) Quantification of TAG liver content. Data were expressed as ±SEM (*n* = 5–8 animals/group and time point). Statistical differences: * *p* < 0.05 compared to CON group (by post hoc Dunn’s multiple comparison test). Abbreviations: *SREBP1C*, sterol regulatory element-binding protein 1c; *FASN*, fatty acid synthase; *ACC1*, acetyl-CoA carboxylase 1; *CPTA1*, carnitine palmitoyl-transferase 1A; *PPARA*, peroxisome proliferator-activated receptor alpha; *CNR1*, cannabinoid receptor 1.

After demonstrating the beneficial influence of EcN and EcoR12 EVs on hepatic inflammation and oxidative stress markers, we examined their impact on lipid metabolism by analyzing the expression of key metabolic enzymes and the transcription factors involved in their regulation in liver samples collected from the three experimental groups. The analysis included genes encoding enzymes involved in fatty acid synthesis, namely acetyl-CoA carboxylase 1 (*ACC1*) and fatty acid synthase (*FASN*), as well as the hepatic transcriptional activator sterol regulatory element-binding protein 1c (*SREBP1C*). For the analysis of the fatty acid oxidation pathway, we selected the gene encoding carnitine palmitoyl-transferase 1A (*CPT1A*), which mediates acyl-CoA transport across the mitochondrial inner membrane, and the regulatory protein peroxisome proliferator-activated receptor alpha (*PPARA*) (Figure 3A). RT-qPCR results showed that EVs from either EcN or EcoR12 significantly downregulated *SREBP1C* and its target genes, *FASN* and *ACC1*, throughout the intervention period (days 8 and 16). Simultaneously, EV treatment, regardless of the producer strain, significantly upregulated genes associated with fatty acid oxidation (*CPT1A* and *PPARA*). Notably, the mRNA levels of the transcriptional activator *PPARA* increased only on day 8, returning to near-control levels with prolonged treatment. In contrast, EV-induced upregulation of *CPT1A* expression was sustained throughout the 16-day treatment period. The above data indicate that orally administered EVs from the beneficial microbiota strains EcN and EcoR12 may influence fatty acid metabolism in the liver, triggering a gene expression profile that favors their oxidation while suppressing the novo synthesis.

In the liver, fatty acid synthesis is also regulated by the endocannabinoid system. Activation of the CB1 receptor (CNR1) influences hepatic fat metabolism by increasing de novo fatty acid synthesis through the upregulation of lipogenic genes [48,49]. Cumulative evidence indicates that CNR1 is upregulated in the liver under steatogenic conditions, such as a high-fat diet and ethanol exposure, and that CNR1 blockade or knockdown exerts anti-lipogenic effects on hepatocytes [50]. Gene expression analysis of *CNR1* in the liver of neonatal rats showed that oral administration of EcN or EcoR12 EVs significantly downregulated the mRNA levels compared to the control group. This regulatory effect was sustained throughout the treatment period (Figure 3A). Notably, this finding aligns with the inhibitory effect exerted by these microbiota EVs on the expression of the regulator SREBP1C and its targets involved in fatty acid synthesis.

To determine whether the anti-lipogenic gene expression profile induced by the administered EVs translated into changes in hepatic lipid content, lipids were extracted from liver homogenates and triglycerides (TAG) were quantified as described in the Methods section. After 8 days of treatment, EVs derived from commensal EcoR12 significantly reduced liver TAG concentrations compared with controls. Furthermore, EVs from the probiotic EcN showed a trend toward reduced TAG levels, although this did not reach statistical significance. After 16 days of treatment, TAG concentrations were comparable across all experimental and control groups (Figure 3B).

The plasma TAG levels were not altered by any of the EV interventions throughout the treatment period (Supplemental Figure S2). This finding suggests that in the absence of liver inflammation and steatosis, the metabolic regulation elicited by microbiota EVs has no impact on normal lipid circulating levels in healthy suckling rats.

To explore other metabolic hepatic effects of the administered EVs we focused on aminotransferases. These are essential enzymes for liver function and are widely recognized as sensitive markers of hepatocellular injury. Since EVs from EcN and EcoR12 have been shown to induce metabolic changes that may protect against chronic liver inflammation and damage, the activities of ALT and AST were assessed in crude liver extracts. In healthy neonatal rats, ALT and AST activity levels remained unchanged following EV interventions (Supplemental Figure S3).

### 3.4. Influence of EcN and EcoR12 EVs on Hepatic Glucose Metabolism During Neonatal Development

Given the profound effects of EVs on lipid-related gene expression reprogramming, we next examined whether vesicle-based interventions could also influence the expression of genes involved in glucose metabolism. This question is particularly relevant in the neonatal period, when glucose metabolism displays distinctive features. During early life, high glucose-6-phosphatase activity ensures a continuous release of glucose into the bloodstream, while glucokinase expression is essentially absent in the first two weeks, thereby restricting hepatic glucose utilization [51,52]. After weaning, dietary glucose and insulin induce glucokinase expression, which reaches adult levels within approximately two additional weeks. This developmental switch secures sufficient glucose supply for the brain while progressively enabling efficient handling of dietary glucose [53].

**Figure 4:**
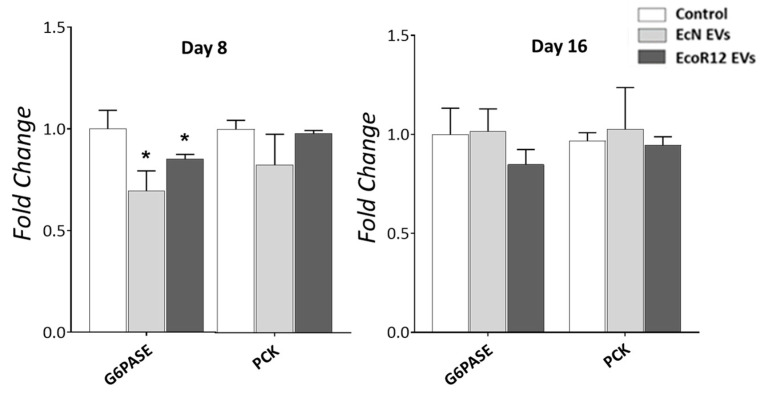
Effects of orally administered EcN and EcoR12 EVs on the expression of genes involved in hepatic glucose production. Neonatal rats were administered with PBS as the control or EVs of the indicated gut microbiota strains (from days 2 to 16 of life). By days 8 and 16, liver samples were collected and processed for RNA isolation. The transcription levels of the indicated genes were quantified by RT-qPCR using *GAPDH* as the reference gene. Relative mRNA levels in the interventional groups were calculated with respect to the CON group (expression value set to 1). Data were expressed as ± SEM (*n* = 5–8 animals/group and time point). Statistical differences: * *p* < 0.05 compared to CON group (by post hoc Dunn’s multiple comparison test). Abbreviations: *PCK*, phosphoenolpyruvate carboxykinase; *G6PASE*, glucose-6-phosphatase.

Within this context, we investigated whether EV administration could modulate the expression of key genes regulating glucose levels. Specifically, we focused on glucokinase (*GCKL*), glucose-6-phosphatase (*G6PASE*) and phosphoenolpyruvate carboxykinase (PCK) of the gluconeogenic pathway. In line with previous reports, we found that the glucokinase gene was not expressed in neonatal rats at any of the examined time points, and EV administration did not alter the absence of *GCKL* expression. Regarding genes involved in hepatic glucose production, EV treatment had no effect on *PCK* expression but led to a downregulation of *G6PASE* mRNA levels on day 8. Notably, this effect was no longer evident by day 16 (Figure 4). Despite the lower *G6PASE* expression at the mRNA level, plasma glucose concentration was not altered by any of the EV interventions (Supplemental Figure S2).

### 3.5. Gene Regulation by EcN or EcoR12 EVs in HepG2 Cells Improves Anti-Inflammatory, Antioxidative, and Lipid Oxidation Pathways

To prove the direct effects of microbiota EVs on hepatocytes, we used the human hepatic cell line HepG2, which is commonly used as an in vitro model to study human liver function and disease. In this study, HepG2 cells were exposed to EVs isolated from the probiotic EcN or the commensal EcoR12 to analyze the expression of genes related to fatty acid metabolism, inflammation and antioxidative pathways. We selected two incubation times, 8 and 24 h, to assess the short- and long-term effects of the treatment.

**Figure 5:**
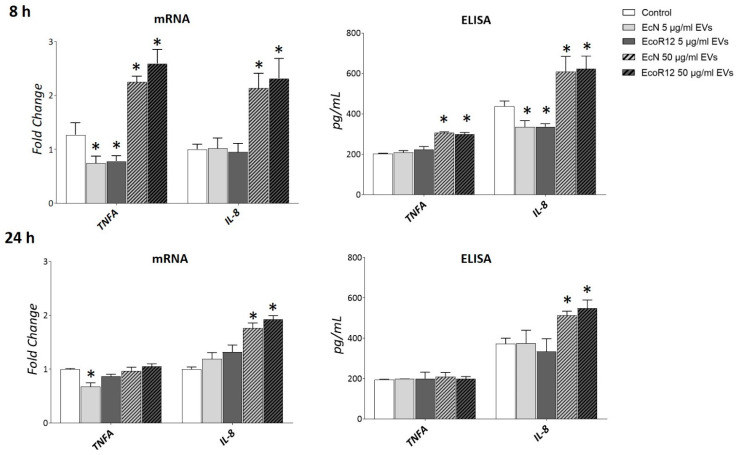
Expression analysis of inflammation markers in HepG2 cells exposed to different doses of bacterial EVs. HepG2 cells were incubated with EVs of the microbiota strains EcN or EcoR12 at 5 μg/mL and 50 μg/m, for 8 or 24 h. The transcription levels of the proinflammatory cytokines TNF-α and IL-8 were assessed by RT-qPCR using *TBP* as the reference gene. Relative mRNA levels were calculated with respect to the CON group (expression value set to 1). Secreted levels of both cytokines were quantified by ELISA in the culture supernatants. In all panels, data were expressed as ±SEM from three independent experiments. Statistical differences: * *p* < 0.05 compared to CON group (by post hoc Tukey’s test).

Initially, we determined the optimal dose of EVs for the experiments. HepG2 cells were incubated with EcN or EcoR12 EVs at two different concentrations, 5 μg/mL and 50 μg/mL, for 8 and 24 h. MTT assays were carried out to assess whether the selected doses were cytotoxic for this hepatic cell line. The results showed that cell viability was not affected by any of them up to 24 h of incubation (Supplemental Figure S4). Additionally, the inflammatory response was chosen as a marker of potential harmful effects. To this end, the expression of genes encoding the proinflammatory cytokines TNF-α and IL-8 was analyzed by RT-qPCR (Figure 5).

At the highest concentration of EVs (50 μg/mL), a significant increase in the mRNA levels of both genes was observed after 8 h of incubation compared to untreated control cells. In contrast, at the lower dose (5 μg/mL), *IL-8* expression levels remained unchanged, while *TNFA* expression was even lower than those of the control. After 24 h of incubation with the higher EV dose, *TNFA* expression returned to control levels, whereas *IL-8* expression remained elevated. Notably, the short-term downregulation of *TNFA* by low doses of EcN EVs in HepG2 cells was consistent with the anti-inflammatory effects observed in rat liver by day 8 of treatment. Quantification of IL-8 and TNF-α secreted levels by ELISA confirmed the inflammation profile of HepG2 cells exposed to a high dose of bacterial EVs (Figure 5). Based on these results we selected the dose of 5 μg/mL EVs for further analysis.

**Figure 6:**
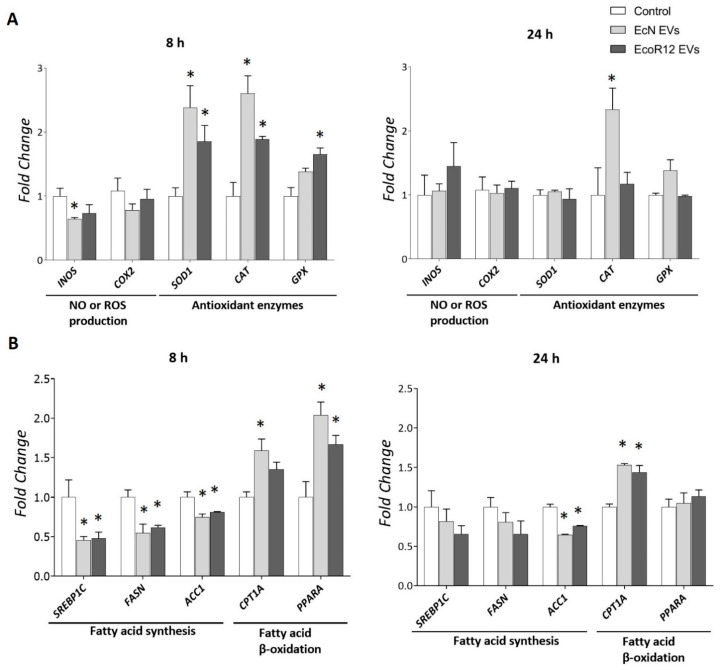
Expression analysis of genes related to oxidative stress (**A**) and lipid metabolism (**B**) in HepG2 cells exposed to EcN or EcoR12 EVs. HepG2 cells were incubated with EVs of the indicated microbiota strains (5 μg/mL) for 8 or 24 h. The transcription levels of the selected genes were assessed by RT-qPCR using *TBP* as the reference gene. Relative mRNA levels were calculated with respect to the CON group (expression value set to 1). In all panels, data were expressed as ±SEM from three independent experiments. Statistical differences: * *p* < 0.05 compared to CON group(by post hoc Tukey’s test). Abbreviations: *INOS*, inducible nitric oxide synthase; *SOD1*, superoxide dismutase; *CAT*, catalase; *GPX*, glutathione peroxidase. *SREBP1C*, sterol regulatory element-binding protein 1c; *FASN*, fatty acid synthase; *ACC1*, acetyl-CoA carboxylase 1; *CPTA1*, carnitine palmitoyl-transferase 1A; *PPARA*, peroxisome proliferator-activated receptor alpha.

The antioxidative effects of EcN and EcoR12 EVs were evaluated by analyzing the expression of genes *SOD1*, *CAT*, *GPX*, *COX2* and *INOS* (Figure 6A). After 8 h of incubation, EVs from both strains significantly upregulated the mRNA levels of the three antioxidant enzymes analyzed. However, this effect was not sustained at longer incubation times, except for catalase. The *CAT* mRNA levels remained elevated in cells treated with EVs from the probiotic strain EcN after 24 h of incubation. Regarding the influence of bacterial EVs on the in vitro generation of oxygen or nitrogen reactive species, *CYP2E1* was not included in the analysis because HepG2 cells exhibit very low or undetectable expression levels of this cytochrome P450 family enzyme [54,55]. In the in vitro HepG2 cell model, the effects of EVs were strain-specific. Consistent with the in vivo results, only EVs from the probiotic EcN reduced *INOS* mRNA levels compared to untreated control cells. This was a short-term effect, as mRNA levels returned to baseline after 24 h. Although differences did not reach statistical significance, EcN EVs also tended to reduce *COX2* expression at 8 h. These findings confirm the antioxidative potential EVs from probiotic and commensal *E. coli* strains by enhancing the antioxidative response in hepatic cells. Additionally, EVs form EcN may protect from oxidative stress by downregulating genes encoding enzymes associated with prooxidant reactive species.

Regarding the impact of gut bacteria-derived EVs on lipid metabolism in HepG2 cells, we analyzed the same genes involved in fatty acid catabolic and anabolic pathways as in the in vivo suckling rat model. The gene *CNR1* was excluded from this in vitro analysis because its Ct values were below the detection limit. This finding was consistent with previous reports in the literature on the low *CNR1* basal expression in HepG2 cells [56]. The results closely aligned with the in vivo data. After 8 h of incubation, EVs from both strains downregulated the expression of lipogenic genes *SREBP1C*, *FASN* and *ACC1* while upregulating the expression of fatty acid oxidation-related genes *CPT1A* and *PPARA* (Figure 6B). However, the modulatory effects of bacterial EVs on the expression of the regulatory genes *SREBP1C* and *PPARA*, as well as the metabolic gene *FASN* were not apparent at longer incubation times. In contrast, the regulation (upregulation or downregulation) of genes encoding the rate-limiting enzymes for lipid oxidation (*CPT1A*) and synthesis (*ACC1*) was sustained throughout 24 h of incubation with EcN or EcoR12 EVs (Figure 6B).

Importantly, the gene expression profile of HepG2 cells stimulated with a higher dose of EVs (50 μg/mL) closely resembled that observed with the lower concentration of 5 µg/mL, except for *CPT1A* (Supplemental Figure S5). This finding suggests that the metabolic regulatory effects of microbiota-derived EVs are achieved at very low concentrations, potentially reflecting physiological conditions in vivo.

Overall, the in vitro results from the direct stimulation of hepatic cells with gut microbiota-derived EVs support the role of these vesicles in modulating liver function through the gut-liver axis.

## 4. Discussion

The gut microbial community plays a crucial role in regulating multiple host functions. Notably, most interactions between the host and the microbiota are indirect, mediated by a wide range of bacterial secreted factors that can enter the bloodstream and reach extraintestinal tissues and organs [23,25,57]. In this context, EVs released by the gut microbiota are emerging as key players in the communication networks linking the gut to distant organs [10,18,58]. Given that microbiota-derived EVs are transported through the systemic circulation and carry bioactive molecules that facilitate cell-to-cell communication, they play a crucial role in maintaining body homeostasis and well-being under healthy conditions, while also contributing to disease progression under dysbiosis and the resulting leaky gut. Despite growing evidence supporting the beneficial properties of microbiota-derived EVs in the gut environment, particularly their role in modulating intestinal barrier integrity and host immune responses, research into their broader systemic effects remains limited. In this context, few studies have investigated the role of microbiota-derived EVs in major inter-organ axes within physiologically healthy models.

The liver is the first organ to receive intestinal metabolites and microbial compounds, absorbed or translocated across the gut epithelial barrier, via the portal vein. The influence of gut beneficial bacteria and their derived EVs on liver function has been primarily studied in cellular and experimental models of fatty liver disease, including metabolic dysfunction-associated fatty liver disease (MAFLD) and alcohol-associated liver disease [24,26,27,59]. These studies revealed that oral administration of EVs derived from *Akkermansia muciniphila*, *Lactobacillus rhamnosus* GG or *Enterococcus faecium* improved intestinal permeability and protected liver against metabolic or alcohol-induced liver injury, fibrosis and metabolic dysfunction, with the effects being dependent on the producing bacterial strain.

The novelty of our study lies in investigating the effects of interventions based on the oral administration of EVs derived from gut-beneficial *E. coli* strains on liver function in healthy neonatal rats. This in vivo model is particularly relevant, as neonatal rodents are born with immature intestinal function and immunity. The high permeability of the neonatal intestine facilitates the transfer of luminal contents into the lamina propria and, subsequently, into the portal vein and systemic circulation. This condition enables the transmission of microbiota-derived compounds and antigens that support the development and education of the newborn immune system, as well as maternal immunoglobulins that confer immune protection to the pups [38]. Therefore, the early postnatal stage represents a critical window for gut development, during which host-microbiota interactions can have a long-lasting impact on health. In this context, the model used in this study enables the investigation of the physiological role of gut microbiota-derived EVs in modulating liver metabolism under healthy conditions, in the absence of liver dysfunction or injury. Previous studies by our group using the same suckling rat model demonstrated the safety and efficacy of interventions based on EVs from the probiotic EcN and the commensal strain EcoR12 in promoting intestinal maturation and enhancing innate and adaptive immune responses during the neonatal period, under both healthy conditions and rotavirus infection [30,31].

The results presented here show that orally administered EVs reach the liver. After normalizing by the total protein concentration in liver samples, elevated LPS levels were observed in treated rats compared to controls, as measured by both ELISA and Western blot. Hepatic LPS content correlated with the presence of the outer membrane protein OmpA, a component of EVs released by Gram-negative bacteria. The basal levels of LPS and OmpA observed in liver samples from control animals likely reflect EVs derived from the indigenous gut microbiota, whereas the elevated levels in treated groups are attributable to the administered EVs. The results from the control group are consistent with previous studies reporting the presence of LPS-containing EVs in the hepatic portal vein and liver tissue of both lean and obese mice [15]. Concerning the fate of administered EVs, other studies have reported their rapid trafficking to the liver [14,60]. Importantly, after seven days of treatment (day 8 of life), plasma zonulin levels were reduced by EVs from either the probiotic EcN or the commensal EcoR12. This finding confirms the efficacy of administered EVs in counteracting the increased intestinal permeability associated with intestinal immaturity in early life and suggests their potential applicability in other conditions characterized by a leaky gut. Despite reducing intestinal permeability, the administered EVs reach the liver, confirming the natural trafficking of EVs released by gut bacteria through the gut-liver axis in healthy individuals.

Since EVs derived from Gram-negative bacterial strains carry LPS and other MAMPs that interact with cell surface and intracellular PRRs, which activate inflammatory signaling cascades [10,61,62], we aimed to assess whether the amount of administered EVs reaching the liver could induce hepatic inflammation or disrupt redox balance. The results revealed strain specific-anti-inflammatory effects. At the administered doses, EVs from the commensal strain EcoR12 did not trigger adverse inflammatory responses, as the expression levels of pro-inflammatory cytokines (IL-12 and TNF-α) and inflammation-associated enzymes (INOS, COX2) remained comparable to those in control animals at both day 8 and day 16. In contrast, EVs from the probiotic EcN induced anti-inflammatory effects by suppressing the expression of these pro-inflammatory cytokines. Notably, this effect was observed only on day 8, corresponding to the phase of intestinal immaturity and enhanced permeability, which is consistent with previous reports describing the anti-inflammatory properties of EcN EVs in vivo and in vitro models of intestinal barrier disruption and inflammation [29,35]. By day 16, *IL12* and *TNFA* mRNA levels were comparable between control and EcN-EV-treated animals; however, the expression of *COX2* and *INOS* showed a decreasing trend in animals receiving EcN EVs. In addition to their effects in the liver, downregulation of both enzymes by EcN EVs in intestinal tissue has previously been reported in experimental models of ulcerative colitis [29]. Inflammation and oxidative stress are closely interconnected processes. The enzymes COX2 and iNOS are important mediators of inflammation, catalyzing the formation of prostaglandin PGE_2_ and nitric oxide, respectively. Additionally, they generate reactive oxygen and nitrogen species that can damage tissues by oxidizing lipids, proteins and DNA. Another major source of ROS in the liver is CYP2E1. This inducible enzyme plays an important role in liver homeostasis, as it is involved in the metabolism of endogenous metabolites such as acetone and fatty acids, as well as xenobiotics. Increasing evidence indicates that the upregulation of *CYP2E1* contributes significantly to the progression of chronic fatty liver diseases [63]. Notably, interventions involving EcN- or EcoR12-derived EVs led to a sustained downregulation of *CYP2E1* throughout the treatment period. This regulation was accompanied by a robust induction of genes encoding key enzymes involved in the cellular antioxidant response, namely *SOD1*, *CAT*, and *GPX*. By reducing hepatic ROS production and enhancing the antioxidant enzyme arsenal, EVs released by microbiota beneficial *E. coli* strains could protect liver from oxidative stress and subsequent tissue damage. In addition, EVs derived from the probiotic EcN may contribute to the attenuation of liver inflammation.

To assess the liver metabolic effects of the administered EVs, we focused on lipid metabolism, specifically examining the expression of genes encoding the rate-limiting enzymes of de novo fatty acid synthesis (ACC1 and FASN) and fatty acid oxidation (CPT1) pathways, as well as their key hepatic transcriptional regulators, SREBP1C and PPARA, respectively. Our results revealed that both EcN and EcoR12 EVs significantly modulate the expression of these genes, downregulating those involved in lipid synthesis while upregulating genes associated with fatty acid degradation. In the liver, CNR1 plays a critical role in regulating lipid metabolism under physiological conditions by upregulating the expression of lipogenic genes involved in fatty acid synthesis. Its expression is influenced by nutritional status and is particularly elevated in fatty liver diseases [50]. Notably, pharmacological inhibition of CNR1 using specific antagonists has been shown to reduce hepatic steatosis, improve lipid profiles and attenuate oxidative stress and proinflammatory cytokine production [50,64]. Consistent with their anti-lipogenic and antioxidant effects, oral administration of either EcN or EcoR12 EVs led to a significant downregulation of CNR1 compared to control animals. Overall, these findings provide evidence that EVs from both beneficial *E. coli* strains influence hepatic lipid metabolism via the gut-liver axis. Specifically, they trigger a gene expression profile that favors fatty acid degradation pathways while suppressing lipid synthesis, thus preventing their subsequent accumulation in the liver. Consequently, liver TAG content was reduced in the groups receiving EVs, particularly EcoR12 EVs, during the first 8 days of life. Similar to other regulated parameters, such as pro-inflammatory cytokines and plasma zonulin levels, the vesicle-mediated effects on TAG accumulation were most evident during the period of neonatal immaturity, then returned to baseline as intestinal maturation and permeability normalized. By day 16, both control and treated groups exhibited comparable hepatic TAG levels; however, EV treatment maintained an anti-lipogenic gene expression profile. In healthy animals, the distribution of nutrients to different metabolic pathways is regulated by the physiological status, diet, and hormonal signaling, primarily through post-translational mechanisms that modulate enzyme activities and metabolite fluxes [65]. In neonatal suckling rats, the nutritional components of maternal milk are preferentially utilized for growth and development rather than being diverted to fat storage. Therefore, despite the favorable gene expression profile, nutrient-derived carbons are not available in excess for accumulation in the liver.

In contrast to the pronounced effects on lipid metabolic gene expression, interventions with EcN or EcoR12 EVs did not significantly alter the neonatal expression of genes involved in hepatic glucose trapping (*GCKL*) or glucose production, except for *G6PASE*. Notably, despite reducing *G6PASE* mRNA levels, EV treatment preserved normoglycemia, comparable to control pups. It is important to note that higher transcription levels do not necessarily correlate with increased expression of the enzyme active form, as post-translational regulatory mechanisms can intervene. Indeed, mechanisms that modulate the accessibility of microsome-associated G6Pase in the liver of neonatal rats have been described. At birth, transcription of the gene is activated, yet a substantial fraction of the enzyme remains sequestered in an inactive state, a process referred to as latency. During maturation, latency decreases over time, allowing the accessibility of the active enzyme [66]. This mechanism provides precise control over the amount of glucose released into the bloodstream, thereby preventing hyperglycemia. This study shows that EVs from both beneficial *E. coli* strains do not disrupt the temporal pattern of *GCKL* and *G6PASE* expression during the first two weeks of life, thus excluding adverse effects related to disturbances in glycemia. Given the great differences between the neonatal and adult periods, we cannot rule out that EcN or EcoR12 EVs could modulate glucose metabolism in adults.

As stated above, the neonatal period represents a critical stage for gut development, during which microbiota-host interactions can have profound and long-term effects on health. In this context, EVs derived from a healthy, balanced microbiota may positively influence metabolic programming across various gut-organ axes by promoting the establishment of healthy metabolic profiles.

Despite inherent differences between the in vivo neonatal rat model and the in vitro human HepG2 cell model, both experimental approaches yielded comparable results. Exposure of HepG2 cells with low doses of EcN EVs or EcoR12 EVs (5 µg/mL) increased antioxidant capacity, suppressed the expression of de novo lipogenic genes, and activated genes involved in fatty acid oxidation. These effects were most prominent at early incubation times. Notably, EVs from these *E. coli* strains of the gut microbiota showed greater efficacy in modulating lipid metabolism in HepG2 cells compared to EVs derived from well-characterized gut beneficial bacteria such as *A. muciniphila*, a species currently receiving significant attention as a next-generation probiotic [67]. In the HepG2 cell model, *A. muciniphila*-derived EVs did not significantly alter *CNR1* expression relative to the control, and upregulation of *PPARA* was only observed at a vesicle concentration of 100 µg/mL, which is 50 times higher than that used in our study with EcN or EcoR12 EVs [68]. The ability of EcN or EcoR12 EVs to exert metabolic regulatory effects at very low concentrations aligns with the number of vesicles that may reach the liver via the portal vein under healthy gut conditions, reinforcing their physiological role as mediators of systemic homeostasis.

Overall, the results of this study demonstrate that microbiota-derived EVs present in the intestinal tract act as regulators of liver oxidative balance and metabolism under physiological conditions in healthy individuals, confirming their role as key mediators of communication in the gut-liver axis. In addition, they provide a basis for the potential application of EVs derived from the probiotic EcN and the commensal strain EcoR12 as postbiotics for preventing chronic liver diseases involving inflammation, steatosis and oxidative stress-induced injury, such as metabolic syndrome. This condition represents a major public health concern and there is currently no effective treatment for the early stages of the disease or for mitigating long-term complications [69,70,71]. Postbiotics, defined as non-viable bacterial components with health benefits, are emerging as a novel class of biotic therapies. Unlike probiotics, postbiotics pose no risk of bacterial translocation and offer enhanced bioavailability of molecules for interaction with host-specific receptors, thereby triggering targeted biological responses [72,73,74,75]. In this context, EVs from beneficial microbes are emerging as innovative tools to prevent and manage metabolic, neurological, and inflammatory diseases across various gut-organ axes [76,77]. Preclinical assays are essential to establish the efficacy of these EVs in experimental models of liver disease prior to their translation into clinical settings.

## 5. Concluding Remarks

This study confirms that, in healthy individuals, gut microbiota-derived EVs play a central role in the communication network between the gut and the liver. The findings provide compelling evidence that oral administration of EVs from the probiotic EcN and the commensal EcoR12 can beneficially modulate liver function through multiple mechanisms. In the gut, EVs from both beneficial *E. coli* strains help reduce intestinal permeability, thereby preventing the translocation of luminal antigens and harmful compounds to the liver. Upon reaching the liver via the portal vein, these EVs influence gene expression, promoting antioxidant and lipid-lowering profiles that protect against oxidative stress and metabolic damage. Additionally, EVs from the probiotic EcN exert anti-inflammatory effects within the liver. The continuous trafficking of EVs released by a balanced and healthy gut microbiota along the gut-liver axis may support homeostatic mechanisms that promote and maintain liver health. This programming may be important during the neonatal period to achieve long-lasting benefits for well-being.

## Data Availability

The datasets generated during this work have been deposited in the CORA repository of the Universitat de Barcelona (https://doi.org/10.34810/data2298) accessed on 20 September 2025. The data will be fully available when the article is published.

## Supplementary Materials

The following supporting information can be downloaded at: https://www.mdpi.com/article/10.3390/nu17193066/s1, Table S1: TaqMan Assays (Applied Biosystems) used to analyze gene expression in the neonatal rat model; Table S2: Nucleotide sequences of primers used to analyze gene expression in rat liver and HepG2 cells (SYBR Green protocol); Figure S1: Interventions with EcN EVs or EcoR12 EVs do not affect body weight or liver weight; Figure S2: Concentration of triglycerides and glucose in plasma samples from control and vesicle-treated neonatal rats collected at the indicated times; Figure S3: ALT and AST enzyme activities in liver of neonatal rats; Figure S4: Cell viability of HepG2 cells exposed to different doses of bacterial EVs; Figure S5: Regulation of genes involved in fatty acid synthesis and oxidation by different doses of bacterial EVs in HepG2 cells.

## References

[1] Jandhyala S.M., Talukdar R., Subramanyam C., Vuyyuru H., Sasikala M., Reddy D.N. (2015). Role of the Normal Gut Microbiota. World J. Gastroenterol..

[2] Adak A., Khan M.R. (2018). An Insight into Gut Microbiota and Its Functionalities. Cell. Mol. Life Sci..

[3] Ahlawat S., Asha N., Sharma K.K. (2021). Gut–Organ Axis: A Microbial Outreach and Networking. Lett. Appl. Microbiol..

[4] Van Hul M., Cani P.D., Petifils C., De Vos W.M., Tilg H., El Omar E.M. (2024). What Defines a Healthy Gut Microbiome?. Gut.

[5] Afzaal M., Saeed F., Shah Y.A., Hussain M., Rabail R., Socol C.T., Hassoun A., Pateiro M., Lorenzo J.M., Rusu A.V. (2022). Human Gut Microbiota in Health and Disease: Unveiling the Relationship. Front. Microbiol..

[6] De Vos W.M., Tilg H., Van Hul M., Cani P.D. (2022). Gut Microbiome and Health: Mechanistic Insights. Gut.

[7] Johansson M.E.V., Sjövall H., Hansson G.C. (2013). The Gastrointestinal Mucus System in Health and Disease. Nat. Rev. Gastroenterol. Hepatol..

[8] Spadoni I., Zagato E., Bertocchi A., Paolinelli R., Hot E., Di Sabatino A., Caprioli F., Bottiglieri L., Oldani A., Viale G. (2015). A Gut-Vascular Barrier Controls the Systemic Dissemination of Bacteria. Science.

[9] Spadoni I., Fornasa G., Rescigno M. (2017). Organ-Specific Protection Mediated by Cooperation between Vascular and Epithelial Barriers. Nat. Rev. Immunol..

[10] Díaz-Garrido N., Badia J., Baldomà L. (2021). Microbiota-Derived Extracellular Vesicles in Interkingdom Communication in the Gut. J. Extracell. Vesicles.

[11] Kaur H., Ali S.A., Yan F. (2022). Interactions between the Gut Microbiota-Derived Functional Factors and Intestinal Epithelial Cells—Implication in the Microbiota-Host Mutualism. Front. Immunol..

[12] Toyofuku M., Schild S., Kaparakis-Liaskos M., Eberl L. (2023). Composition and Functions of Bacterial Membrane Vesicles. Nat. Rev. Microbiol..

[13] Stentz R., Carvalho A.L., Jones E.J., Carding S.R. (2018). Fantastic Voyage: The Journey of Intestinal Microbiota-Derived Microvesicles through the Body. Biochem. Soc. Trans..

[14] Jones E.J., Booth C., Fonseca S., Parker A., Cross K., Miquel-Clopés A., Hautefort I., Mayer U., Wileman T., Stentz R. (2020). The Uptake, Trafficking, and Biodistribution of Bacteroides Thetaiotaomicron Generated Outer Membrane Vesicles. Front. Microbiol..

[15] Jain H., Kumar A., Almousa S., Mishra S., Langsten K.L., Kim S., Sharma M., Su Y., Singh S., Kerr B.A. (2024). Characterisation of LPS+ Bacterial Extracellular Vesicles along the Gut-Hepatic Portal Vein-Liver Axis. J. Extracell. Vesicles.

[16] Schaack B., Hindré T., Quansah N., Hannani D., Mercier C., Laurin D. (2022). Microbiota-Derived Extracellular Vesicles Detected in Human Blood from Healthy Donors. Int. J. Mol. Sci..

[17] Verbunt J., Jocken J., Blaak E., Savelkoul P., Stassen F. (2024). Gut-Bacteria Derived Membrane Vesicles and Host Metabolic Health: A Narrative Review. Gut Microbes.

[18] Margutti P., D’Ambrosio A., Zamboni S. (2024). Microbiota-Derived Extracellular Vesicle as Emerging Actors in Host Interactions. Int. J. Mol. Sci..

[19] Pabst O., Hornef M.W., Schaap F.G., Cerovic V., Clavel T., Bruns T. (2023). Gut–Liver Axis: Barriers and Functional Circuits. Nat. Rev. Gastroenterol. Hepatol..

[20] Tripathi A., Debelius J., Brenner D.A., Karin M., Loomba R., Schnabl B., Knight R. (2018). Publisher Correction: The Gut–Liver Axis and the Intersection with the Microbiome. Nat. Rev. Gastroenterol. Hepatol..

[21] Martín-Mateos R., Albillos A. (2021). The Role of the Gut-Liver Axis in Metabolic Dysfunction-Associated Fatty Liver Disease. Front. Immunol..

[22] Milosevic I., Vujovic A., Barac A., Djelic M., Korac M., Spurnic A.R., Gmizic I., Stevanovic O., Djordjevic V., Lekic N. (2019). Gut-Liver Axis, Gut Microbiota, and Its Modulation in the Management of Liver Diseases: A Review of the Literature. Int. J. Mol. Sci..

[23] Albillos A., de Gottardi A., Rescigno M. (2020). The Gut-Liver Axis in Liver Disease: Pathophysiological Basis for Therapy. J. Hepatol..

[24] Rao Y., Kuang Z., Li C., Guo S., Xu Y., Zhao D., Hu Y., Song B., Jiang Z., Ge Z. (2021). Gut Akkermansia Muciniphila Ameliorates Metabolic Dysfunction-Associated Fatty Liver Disease by Regulating the Metabolism of L-Aspartate via Gut-Liver Axis. Gut Microbes.

[25] Hsu C.L., Schnabl B. (2023). The Gut–Liver Axis and Gut Microbiota in Health and Liver Disease. Nat. Rev. Microbiol..

[26] Gu Z., Li F., Liu Y., Jiang M., Zhang L., He L., Wilkey D.W., Merchant M., Zhang X., Deng Z.B. (2021). Exosome-Like Nanoparticles From Lactobacillus Rhamnosus GG Protect Against Alcohol-Associated Liver Disease Through Intestinal Aryl Hydrocarbon Receptor in Mice. Hepatol. Commun..

[27] Keshavarz Azizi Raftar S., Ashrafian F., Yadegar A., Lari A., Moradi H.R., Shahriary A., Azimirad M., Alavifard H., Mohsenifar Z., Davari M. (2021). The Protective Effects of Live and Pasteurized Akkermansia Muciniphila and Its Extracellular Vesicles against HFD/CCl4-Induced Liver Injury. Microbiol. Spectr..

[28] Olivo-Martínez Y., Bosch M., Badia J., Baldomà L. (2023). Modulation of the Intestinal Barrier Integrity and Repair by Microbiota Extracellular Vesicles through the Differential Regulation of Trefoil Factor 3 in LS174T Goblet Cells. Nutrients.

[29] Fábrega M.J., Rodríguez-Nogales A., Garrido-Mesa J., Algieri F., Badía J., Giménez R., Gálvez J., Baldomà L. (2017). Intestinal Anti-Inflammatory Effects of Outer Membrane Vesicles from Escherichia Coli Nissle 1917 in DSS-Experimental Colitis in Mice. Front. Microbiol..

[30] Martínez-Ruiz S., Olivo-Martínez Y., Cordero C., Rodríguez-Lagunas M.J., Pérez-Cano F.J., Badia J., Baldoma L. (2024). Microbiota-Derived Extracellular Vesicles as a Postbiotic Strategy to Alleviate Diarrhea and Enhance Immunity in Rotavirus-Infected Neonatal Rats. Int. J. Mol. Sci..

[31] Martínez-Ruiz S., Sáez-Fuertes L., Casanova-Crespo S., Rodríguez-Lagunas M.J., Pérez-Cano F.J., Badia J., Baldoma L. (2023). Microbiota-Derived Extracellular Vesicles Promote Immunity and Intestinal Maturation in Suckling Rats. Nutrients.

[32] Ochman H., Selander R.K. (1984). Standard Reference Strains of Escherichia Coli from Natural Populations. J. Bacteriol..

[33] Diaz-Garrido N., Badia J., Baldomà L. (2022). Modulation of Dendritic Cells by Microbiota Extracellular Vesicles Influences the Cytokine Profile and Exosome Cargo. Nutrients.

[34] Reeves P.G., Nielsen F.H., Fahey G.C. (1993). AIN-93 Purified Diets for Laboratory Rodents: Final Report of the American Institute of Nutrition Ad Hoc Writing Committee on the Reformulation of the AIN-76A Rodent Diet. J. Nutr..

[35] Olivo-Martínez Y., Martínez-Ruiz S., Cordero-Alday C., Bosch M., Badia J., Baldoma L. (2024). Modulation of Serotonin-Related Genes by Extracellular Vesicles of the Probiotic Escherichia Coli Nissle 1917 in the Interleukin-1β-Induced Inflammation Model of Intestinal Epithelial Cells. Int. J. Mol. Sci..

[36] Livak K.J., Schmittgen T.D. (2001). Analysis of Relative Gene Expression Data Using Real-Time Quantitative PCR and the 2-ΔΔCT Method. Methods.

[37] Menon R., Khanipov K., Radnaa E., Ganguly E., Bento G.F.C., Urrabaz-Garza R., Kammala A.K., Yaklic J., Pyles R., Golovko G. (2023). Amplification of Microbial DNA from Bacterial Extracellular Vesicles from Human Placenta. Front. Microbiol..

[38] Weström B., Arévalo Sureda E., Pierzynowska K., Pierzynowski S.G., Pérez-Cano F.J. (2020). The Immature Gut Barrier and Its Importance in Establishing Immunity in Newborn Mammals. Front. Immunol..

[39] Tulkens J., Vergauwen G., Van Deun J., Geeurickx E., Dhondt B., Lippens L., De Scheerder M.A., Miinalainen I., Rappu P., De Geest B.G. (2020). Increased Levels of Systemic LPS-Positive Bacterial Extracellular Vesicles in Patients with Intestinal Barrier Dysfunction. Gut.

[40] Chakaroun R.M., Massier L., Kovacs P. (2020). Gut Microbiome, Intestinal Permeability, and Tissue Bacteria in Metabolic Disease: Perpetrators or Bystanders?. Nutrients.

[41] Zhao C., Xiao C., Feng S., Bai J. (2023). Artemisitene Alters LPS-Induced Oxidative Stress, Inflammation and Ferroptosis in Liver Through Nrf2/HO-1 and NF-KB Pathway. Front. Pharmacol..

[42] Di Vincenzo F., Del Gaudio A., Petito V., Lopetuso L.R., Scaldaferri F. (2024). Gut Microbiota, Intestinal Permeability, and Systemic Inflammation: A Narrative Review. Intern. Emerg. Med..

[43] Del Campo J.A., Gallego P., Grande L. (2018). Role of Inflammatory Response in Liver Diseases: Therapeutic Strategies. World J. Hepatol..

[44] Rodríguez-Nogales A., Algieri F., Garrido-Mesa J., Vezza T., Utrilla M.P., Chueca N., Fernández-Caballero J.A., García F., Rodríguez-Cabezas M.E., Gálvez J. (2018). The Administration of Escherichia Coli Nissle 1917 Ameliorates Development of DSS-Induced Colitis in Mice. Front. Pharmacol..

[45] Park J., Kim D.H., Kim S., Ma H.W., Park I.S., Son M., Kim J.H., Shin Y., Kim S.W., Cheon J.H. (2021). Anti-Inflammatory Properties of Escherichia Coli Nissle 1917 in a Murine Colitis Model. Intest. Res..

[46] Yang L., Roh Y.S., Song J., Zhang B., Liu C., Loomba R., Seki E. (2014). Transforming Growth Factor Beta Signaling in Hepatocytes Participates in Steatohepatitis through Regulation of Cell Death and Lipid Metabolism in Mice. Hepatology.

[47] Fabregat I., Moreno-Càceres J., Sánchez A., Dooley S., Dewidar B., Giannelli G., ten Dijke P. (2016). TGF-β Signalling and Liver Disease. FEBS J..

[48] De Gottardi A., Spahr L., Ravier-Dall’Antonia F., Hadengue A. (2010). Cannabinoid Receptor 1 and 2 Agonists Increase Lipid Accumulation in Hepatocytes. Liver Int..

[49] Shrestha N., Cuffe J.S.M., Hutchinson D.S., Headrick J.P., Perkins A.V., McAinch A.J., Hryciw D.H. (2018). Peripheral Modulation of the Endocannabinoid System in Metabolic Disease. Drug Discov. Today.

[50] Bazwinsky-Wutschke I., Zipprich A., Dehghani F. (2019). Endocannabinoid System in Hepatic Glucose Metabolism, Fatty Liver Disease, and Cirrhosis. Int. J. Mol. Sci..

[51] Chatelain F., Pégorier J.P., Minassian C., Bruni N., Tarpin S., Girard J., Mithieux G. (1998). Development and regulation of glucose-6-phosphatase gene expression in rat liver, intestine, and kidney: In vivo and in vitro studies in cultured fetal hepatocytes. Diabetes.

[52] Walker D.G., Holland G. (1965). The development of hepatic glucokinase in the neonatal rat. Biochem. J..

[53] Walker D.G., Eaton S.W. (1967). Regulation of development of hepatic glucokinase in the neonatal rat by the diet. Biochem. J..

[54] Wu D., Cederbaum A.I. (2000). Ethanol and Arachidonic Acid Produce Toxicity in Hepatocytes from Pyrazole-Treated Rats with High Levels of CYP2E1. Mol. Cell. Biochem..

[55] Killingsworth Z.K., Misare K.R., Ryan A.S., Ampolini E.A., Mendenhall T.T., Engevik M.A., Hartman J.H. (2024). Subcellular Expression of CYP2E1 in HepG2 Cells Impacts Response to Free Oleic and Palmitic Acid. Curr. Res. Toxicol..

[56] Shi D., Zhan X., Yu X., Jia M., Zhang Y., Yao J., Hu X., Bao Z. (2014). Inhibiting CB1 Receptors Improves Lipogenesis in an in Vitro Non-Alcoholic Fatty Liver Disease Model. Lipids Health Dis..

[57] Anand S., Mande S.S. (2022). Host-Microbiome Interactions: Gut-Liver Axis and Its Connection with Other Organs. NPJ Biofilms Microbiomes.

[58] Wang S., Luo J., Wang H., Chen T., Sun J., Xi Q., Zhang Y. (2024). Extracellular Vesicles: A Crucial Player in the Intestinal Microenvironment and Beyond. Int. J. Mol. Sci..

[59] Zhu Y., Zhang X., Huang W.H., Luo M., Feng X., Zhang H., Qi Q. (2025). Protective Effect of Enterococcus Faecium Against Alcohol-Induced Acute Liver Injury Via Extracellular Vesicles in Rats. Foodborne Pathog. Dis..

[60] Cohen S.J., Meyerovich G., Blank S., Ovdat E., Loewenstein S., Kania-Almog J., Cohen M., Lahat G., Klausner J.M., Lubezky N. (2023). Microbiota Transfer Following Liver Surgery Involves Microbial Extracellular Vesicle Migration That Affects Liver Immunity. Hepatol. Commun..

[61] Kaparakis-Liaskos M., Ferrero R.L. (2015). Immune Modulation by Bacterial Outer Membrane Vesicles. Nat. Rev. Immunol..

[62] Liu B.D., Akbar R., Oliverio A., Thapa K., Wang X., Fan G.C. (2024). Bacteria Extracellular Vesicles in the Regulation of Inflammatory Response and Host-Microbe Interactions. Shock.

[63] Massart J., Begriche K., Hartman J.H., Fromenty B. (2022). Role of Mitochondrial Cytochrome P450 2E1 in Healthy and Diseased Liver. Cells.

[64] Jorgačević B., Vučević D., Samardžić J., Mladenović D., Vesković M., Vukićević D., Ješić R., Radosavljević T. (2020). The Effect of CB1 Antagonism on Hepatic Oxidative/Nitrosative Stress and Inflammation in Nonalcoholic Fatty Liver Disease. Curr. Med. Chem..

[65] Pignatti C., D’Adamo S., Flamigni F., Cetrullo S. (2020). Molecular Mechanisms Linking Nutrition to Metabolic Homeostasis: An Overview Picture of Current Understanding. Crit. Rev. Eukaryot. Gene Expr..

[66] Burchell A., Leakey J.E. (1988). Development of the rat hepatic microsomal glucose-6-phosphatase system and its glucocorticoid inducibility. Biol. Neonate.

[67] De Filippis F., Esposito A., Ercolini D. (2022). Outlook on Next-Generation Probiotics from the Human Gut. Cell. Mol. Life Sci..

[68] Ghaderi F., Sotoodehnejadnematalahi F., Hajebrahimi Z., Fateh A., Siadat S.D. (2022). Effects of Active, Inactive, and Derivatives of Akkermansia Muciniphila on the Expression of the Endocannabinoid System and PPARs Genes. Sci. Rep..

[69] Miao L., Targher G., Byrne C.D., Cao Y.Y., Zheng M.H. (2024). Current Status and Future Trends of the Global Burden of MASLD. Trends Endocrinol. Metab..

[70] Huang D.Q., Wong V.W.S., Rinella M.E., Boursier J., Lazarus J.V., Yki-Järvinen H., Loomba R. (2025). Metabolic Dysfunction-Associated Steatotic Liver Disease in Adults. Nat. Rev. Dis. Primers.

[71] Seitz H.K., Bataller R., Cortez-Pinto H., Gao B., Gual A., Lackner C., Mathurin P., Mueller S., Szabo G., Tsukamoto H. (2018). Alcoholic Liver Disease. Nat. Rev. Dis. Primers.

[72] Salminen S., Collado M.C., Endo A., Hill C., Lebeer S., Quigley E.M.M., Sanders M.E., Shamir R., Swann J.R., Szajewska H. (2021). The International Scientific Association of Probiotics and Prebiotics (ISAPP) Consensus Statement on the Definition and Scope of Postbiotics. Nat. Rev. Gastroenterol. Hepatol..

[73] Wegh C.A.M., Geerlings S.Y., Knol J., Roeselers G., Belzer C. (2019). Postbiotics and Their Potential Applications in Early Life Nutrition and Beyond. Int. J.Mol. Sci..

[74] Żółkiewicz J., Marzec A., Ruszczyński M., Feleszko W. (2020). Postbiotics—A Step Beyond Pre- and Probiotics. Nutrients.

[75] Ma L., Tu H., Chen T. (2023). Postbiotics in Human Health: A Narrative Review. Nutrients.

[76] Xie J., Li Q., Haesebrouck F., Van Hoecke L., Vandenbroucke R.E. (2022). The Tremendous Biomedical Potential of Bacterial Extracellular Vesicles. Trends Biotechnol..

[77] Xie J., Li Q., Nie S. (2024). Bacterial Extracellular Vesicles: An Emerging Postbiotic. Trends Food. Sci. Technol..

