# Supplementary material for: Extracellular Vesicles from Escherichia coli Strains of the Gut Microbiota Trigger Hepatic Antioxidant and Anti-Lipogenic Effects via the Gut-Liver Axis in Healthy Neonatal Rats
Source: Nutrients. 2025 Sep 25;17(19):3066. doi: 10.3390/nu17193066 (PMC12525914; doi:10.3390/nu17193066)
Supplement: Supplementary file 1 [file nutrients-17-03066-s001.zip › nutrients-3801294-Supplemental material-R1.pdf]

Table S1. TaqMan Assays (Applied Biosystems) used to analyze gene expression in the neonatal rat model.

Gene	reference
PPARG	Rn00440945_m1
PPARA	Rn00566193_m1
ACC1	Rn00573474_m1
GUSB	Rn00566655_m1
CPT1	Rn00580702_m1
FASN	Rn00569117_m1
SREBP1c	Rn01495763_g1
IL12	Rn00584538_m1
TNFA	Rn99999017_m1
SOD	Rn00566938_m1
CAT	Rn00560930_m1
GPX	Rn00577994_g1
COX2	Rn01483828_m1
iNOS	Rn00561646_m1
CYP2E1	Rn00580624_m1

Table S2. Nucleotide sequences of primers used to analyze gene expression in rat liver and HepG2 cells (SYBR Green protocol).

Gene	Forward Sequence (5'-3')	Reverse Sequence (5'-3')	Organism
PPARG	GGGATCAGCTCCGTGGATCT	TGCACTTTGGTACTCTTGAAGTT	Human
PPARA	CGGTGACTTATCCTGTGGTCC	CCGCAGATTCTACATTCGATGTT	Human
ACC1	CATGCGGTCTATCCGTAGGTG	GTGTGACCATGACAACGAATCT	Human
TBP	GTTCAGCAGTCAACGTCCCA	TCATGGGGGAGGGATACAGT	Human
CPT1	TTTCCTTGCTGAGGTGCTCT	TCTCGCCTGCAATCATGTAG	Human
FASN	TCGTGGGCTACAGCATGGT	GCCCTCTGAAGTCGAAGAAGAA	Human
SREBP1c	CGGAGCCATGGATTGCACT	TAGGCCAGGGAAGTCACTG	Human
IL8	CTGGCCGTGGCTCTCTTG	GGGTGGAAAGGTTTGGAGTATG	Human
TNFA	AACTAGTGGTGCCAGCCGAT	CTTCACAGAGCAATGACTCC	Human
SOD	ATCCTCTATCCAGAAAACACG	ACACCACAAGCCAAACGAC	Human
CAT	TGTTGAAGATGCGGCGAG	ATGAGAGGGTAGTCCTTGTG	Human
GPX	GCCTTCCCGTGTAACCAGT	GCGAACTCTTTGATCTCTTCGT	Human
COX2	GGGTTGCTGGGGGAAGAAATG	GGTGGCTGTTTTGGTAGGCTG	Human
INOS	GTTGAAGACTGAGACTCTGG	ACTAGGCTACTCCGTGGA	Human
GAPDH	TGTGTTGTCCCTGTATGCCTCT	AAAACGCAGCTCAGTAACAGTCC	Rat
G6PASE	GACCTCAGGAACGCCTTCTATG	ATTGATGCCCACAGTCTCTTGA	Rat
PCK	GTCACCATCACTTCCTGGAAGA	GGTGCAGAATCGCGAGTTG	Rat
GCK	TGGCCTAATGAAAGCTGGGG	AGCACCCTGTTCCATACGTG	Rat

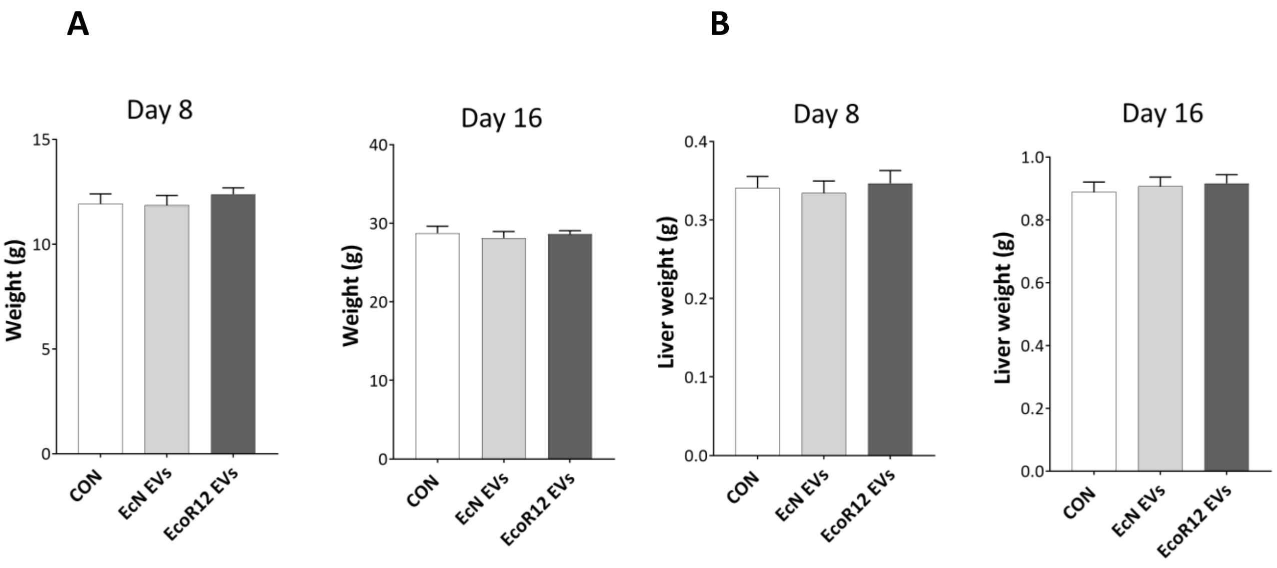

Figure S1. Interventions with EcN EVs or EcoR12 EVs do not affect body weight (A) or liver weight (B). The experimental groups were: Control (white bars), EcN-EVs (light gray bars) and EcoR12-EVs (dark gray bars). Results are expressed as mean ± SEM (n=12 animals/group). No significant differences were observed between groups.

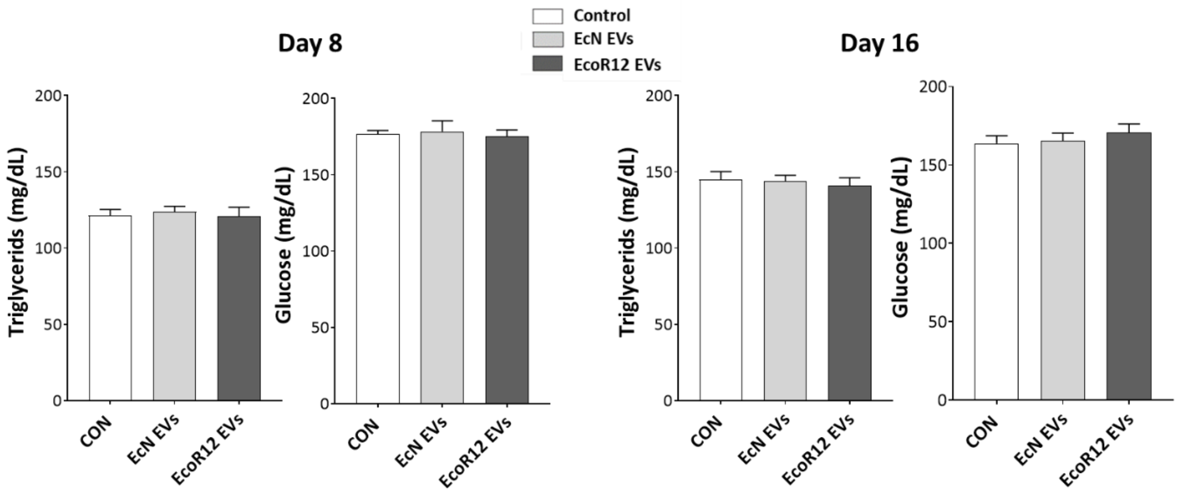

Figure S2. Concentration of triglycerides and glucose in plasma samples from control and vesicle-treated neonatal rats collected at the indicated times. Neonatal rats were administered PBS as the control or EVs of the indicated gut microbiota strains (from days 2 to 16 of life). By days 8 and 16, blood samples were collected and processed for plasma obtention. No statistical differences were observed by the post hoc Dunn's multiple comparison test.

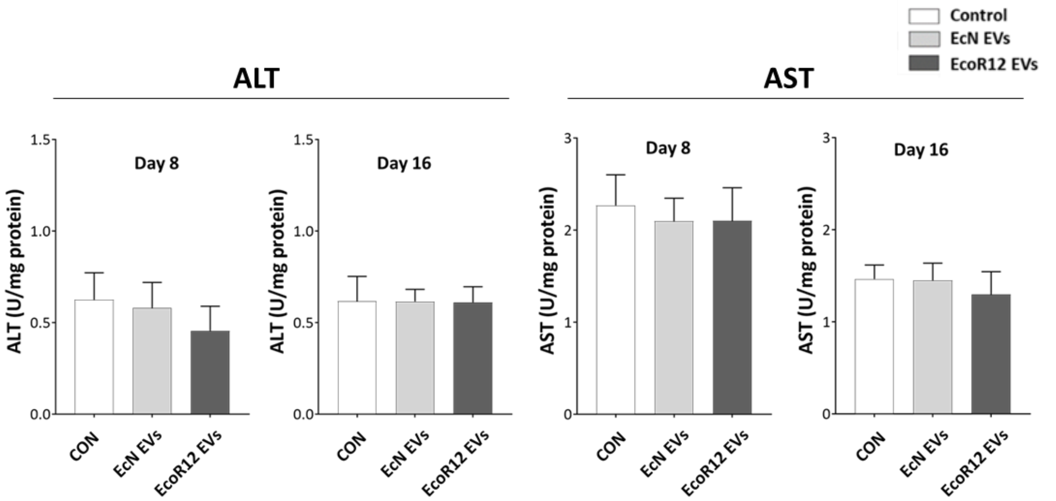

Figure S3. ALT and AST enzyme activities in liver of neonatal rats. Neonatal rats were administered EVs of the indicated strains (from days 2 to 16 of life). The control group received PBS. By days 8 and 16, liver samples were collected and processed to obtain crude extracts. Alanine aminotransferase (ALT) and aspartate aminotransferase (AST) activities were measured using colorimetric reaction-based kits. No statistical differences were observed between groups.

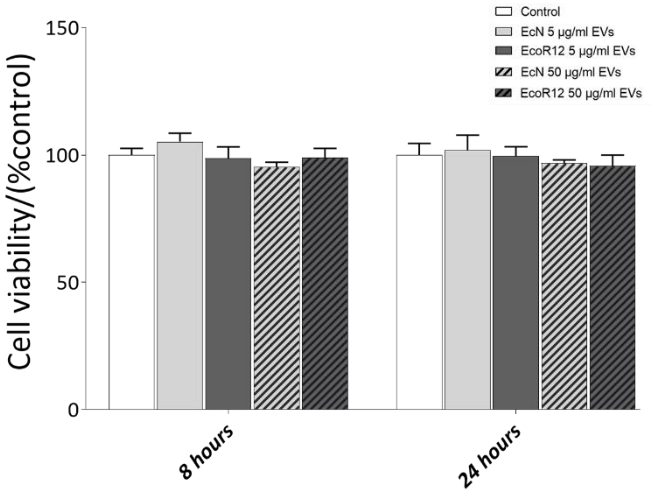

Figure S4. Cell viability of HepG2 cells exposed to different doses of bacterial EVs. HepG2 cells were exposed to EVs of the microbiota strains EcN or EcoR12 at 5 µg/mL and 50 µg/mL, for 8 h or 24 h. Cell viability was assessed by the MTT assays. Data were expressed as percentage of viability when compared to untreated control cells (value set to 100%). No statistical differences were observed by the post hoc Tukey's test.

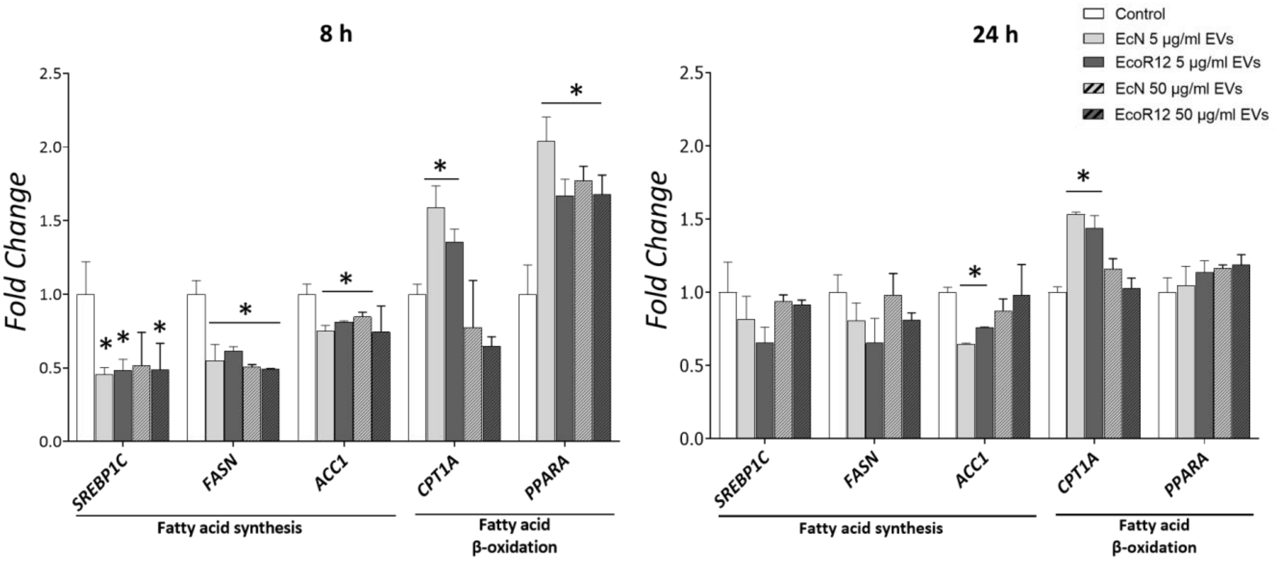

Figure S5. Regulation of genes involved in fatty acid synthesis and oxidation by different doses of bacterial EVs in HepG2 cells. HepG2 cells were incubated with EVs of the microbiota strains EcN or EcoR12 at 5 µg/mL and 50 µg/mL, for 8 h or 24 h. The transcription levels of the indicated genes were measured by RT-qPCR using TBP as the reference gene. Relative mRNA levels were calculated with respect to the CON group (expression value set to 1). Data were expressed as ± SEM from three independent experiments. Statistical differences: * $p < 0.05$ compared to CON group (by post hoc Tukey's test). Abbreviations: *SREBP1c*, sterol regulatory element-binding protein 1c; *FASN*, fatty acid synthase; *ACC1*, acetyl-CoA carboxylase 1; *CPTA1*, carnitine palmitoyl-transferase 1A; *PPARA,* peroxisome proliferator-activated receptor alpha; *CNR1*, cannabinoid receptor 1.
